# Exploring the Gender Preferences for Healthcare Providers and Their Influence on Patient Satisfaction

**DOI:** 10.3390/healthcare13091063

**Published:** 2025-05-05

**Authors:** Felician Andrew Kitole, Zaiba Ali, Jiayi Song, Muhammad Ali, Mochammad Fahlevi, Mohammed Aljuaid, Petra Heidler, Muhammad Ali Yahya, Muhammad Shahid

**Affiliations:** 1 Department of Economics, Mzumbe University, Morogoro P.O. Box 5, Tanzania; felicianandrew@gmail.com; 2 Department of Management, Barkatullah University, Bhopal 462026, India; zaibaali1612@gmail.com; 3 School of Healthcare Management, Tsinghua University, Beijing 100084, China; songjiay22@mails.tsinghua.edu.cn; 4 Department of Economics, Al-Madinah International University, Al-Madinah 57100, Malaysia; alimuhammad1447@gmail.com; 5 Management Department, BINUS Online, Bina Nusantara University, Jakarta 11480, Indonesia; mochammad.fahlevi@binus.ac.id; 6 Operation Research and Management Sciences, Faculty of Business and Management, University Sultan Zainal Abidin, Kampung Gong Badak 21300, Malaysia; 7 Department of Health Administration, College of Business Administration, King Saud University, Riyadh 11451, Saudi Arabia; maljuaid@ksu.edu.sa; 8 Institute of International Trade and Sustainable Economy, IMC University of Applied Sciences, 3500 Krems, Austria; petra.heidler@fh-krems.ac.at; 9 Department of Artificial Intelligence, School of Computing, The Islamia University of Bahawalpur, Bahawalpur 63100, Pakistan; f23barin1m01131@iub.edu.pk; 10 School of Insurance and Economics, University of International Business and Economics, Beijing 100029, China

**Keywords:** patient satisfaction, patient-centered care, equitable healthcare delivery, gender preferences, health equity

## Abstract

**Background:** Patient satisfaction is a key indicator for improving healthcare delivery, yet the influence of gender preferences on healthcare providers remains underexplored. Cultural norms and gender perceptions often shape the patient preferences, affecting access to care, patient–provider relationships, and overall satisfaction. Thus, this study investigates the patients’ gender preferences and their impact on satisfaction in Tanzania. **Methods:** The study utilized a cross-sectional design, collecting data from five health centres: Mikongeni, Konga, Mzumbe, Tangeni, and Mlali. A total of 240 randomly selected respondents participated in the study. Gender preferences were categorized as male, female, and both, and determinants were analyzed using a multivariate probit model (MPM), while satisfaction was analyzed using an ordered logit model (OLM). **Results:** Results reveal that female providers were preferred for empathy (58.30%), intimate care (50.00%), and receptionist roles (50.00%), while males were favored for surgery (50.00%), professionalism (0.86), and IT roles (41.70%). Professionalism (0.75) and communication (0.70) had the strongest positive effects on very high satisfaction. Male provider preference was strongly linked to higher satisfaction (0.84), while female preference showed a mild effect (0.23). Insurance (0.32) and care at Tangeni Health Centre (0.70) boosted satisfaction, while consultation fees (−0.26) reduced it. **Conclusions:** The study recommends that healthcare systems address gender stereotypes by equipping all providers with both technical and relational care skills, regardless of gender. It also highlights the need for culturally and religiously sensitive care practices that acknowledge how societal norms shape patient preferences and satisfaction. To enhance patient-centered care, policies should promote affordability, broaden insurance coverage, and integrate patient feedback on gender preferences into healthcare delivery models.

## 1. Introduction

Healthcare plays a fundamental role in improving the overall well-being of individuals and societies, serving as the foundation for human development and economic growth [1,2]. It encompasses a range of services to promote health, prevent diseases, and manage various conditions, ensuring that individuals have access to quality care when needed [3,4]. Beyond its clinical objectives, healthcare contributes to societal stability by reducing the burden of illness, enabling individuals to contribute productively to their communities, and improving life expectancy [4]. The healthcare sector also functions as a safety net, supporting vulnerable and marginalized populations [5]. Its impact extends beyond individual patients, influencing families, communities, and nations [6]. This critical role highlights the need for effective and inclusive healthcare delivery models that meet the diverse needs of all individuals [7].

The relationship and interaction between patients and healthcare providers are pivotal to delivering high-quality care [8,9]. These transactional interactions form the basis of a therapeutic alliance that fosters trust and cooperation [10]. Healthcare providers act as both caregivers and guides, offering medical expertise while addressing patients’ concerns and emotional needs [11]. Open and transparent communication is a cornerstone of this relationship, allowing providers to explain diagnoses, discuss treatment options, and engage patients in shared decision making [11,12]. This collaborative approach ensures that patients feel empowered and involved in their care, which is critical for achieving positive health outcomes [13]. Strong interpersonal relationships between providers and patients have been shown to improve adherence to medical advice and increase patient satisfaction, ultimately enhancing the efficacy of healthcare services [14].

Comfort and trust significantly influence a patient’s experience in the healthcare system [15]. When patients feel at ease with their providers, they are more likely to disclose personal and sensitive information that is essential for accurate diagnoses and effective treatment plans [16]. In this context, trust goes beyond the provider’s medical expertise and extends to their demeanor, empathy, and respect for the patient’s individuality [17]. For instance, patients who perceive their providers as attentive and respectful are more inclined to follow prescribed treatments and attend follow up visits [18]. Furthermore, the cultural competence of healthcare providers, including their ability to understand and respect cultural differences, plays a vital role in fostering comfort, particularly in multicultural societies [19]. However, beyond these interpersonal factors, patients’ trust is also deeply rooted in perceptions of technical competence. Numerous studies have shown that patients are more likely to trust and follow providers they believe are clinically skilled, accurate in diagnosis, and capable in decision making [14,18,19]. Competence is often evaluated through both observable skills, such as confidence during examinations and clarity in explaining procedures, and contextual cues like provider credentials, experience, and affiliation with reputable institutions. When patients perceive a high level of clinical competence, they are more assured of the provider’s ability to deliver safe, reliable, and effective care, which significantly strengthens the trust relationship [20]. This underlines that trust is not only emotional or relational, but also rational and grounded in evidence of provider capability. Building such trust requires both technical competence and strong interpersonal skills, including active listening and genuine concern for the patient’s well-being [20].

Patient preferences for specific provider characteristics, including gender, add another layer of complexity to the patient–provider relationship [21]. These preferences often blend cultural norms, societal expectations, and individual experiences [22]. For example, some patients may feel more comfortable discussing sensitive health issues, such as sexual or reproductive health, with providers of the same gender [23]. Conversely, in particular cultural or professional contexts, male providers might be perceived as more authoritative or skilled in specific specialties, influencing patient preferences [24]. In developed countries, studies have often found that patients prioritize attributes like empathy, communication, and professionalism over provider gender, indicating a shift toward patient-centered care [23,25]. However, gender preferences remain particularly pronounced in intimate examinations or culturally sensitive issues [25].

In developing countries, cultural and societal factors play a more prominent role in shaping patient preferences for provider gender [24]. For instance, in conservative societies, female patients may insist on female providers for gynecological or obstetric care due to cultural norms surrounding modesty [26]. Similarly, patriarchal structures in some regions may lead to a preference for male providers, particularly in leadership roles or complex specialties [27,28]. These preferences highlight the need to contextualize gender dynamics within the broader cultural and social landscape [29]. Despite these variations, there is a shared recognition across both developed and developing nations that accommodating patient preferences, including gender, enhances patient satisfaction and trust in the healthcare system [30].

Although research on gender preferences in healthcare exists, it remains geographically fragmented and often limited to specific medical specialties, making it difficult to draw broad conclusions [31,32]. Furthermore, many studies overlook emerging trends such as digital health, which may change traditional gender dynamics by reducing direct physical interactions between patients and providers [33]. These gaps highlight the need to better understand how gender preferences affect patient satisfaction, particularly in diverse and changing healthcare environments [31,34]. To address these limitations, this study explores gender preferences in healthcare providers and their influence on patient satisfaction in the rural areas of the Mvomero District, Morogoro Region, Tanzania.

## 2. Method and Materials

### 2.1. Theoretical Framework

This study is grounded in two complementary theories: patient-centered care (PCC) theory and social role theory. These frameworks provide a wide-ranging lens to understand the factors influencing patient preferences for the gender of healthcare providers and their impact on patient satisfaction. Scholars such as Stewart [33], and Mead and Bower [34] popularized the patient-centered care theory. PCC emphasizes treating patients as active participants in their care, recognizing their unique needs, values, and preferences [35,36,37,38]. At its core, PCC advocates for a holistic approach to healthcare, where providers respect and prioritize patients’ individual experiences and preferences. It includes fostering open communication, building trust, and ensuring care is tailored to each patient’s circumstances [39,40].

PCC is highly relevant to this study because it frames patient preferences as a critical component of quality care. Gender preferences, whether shaped by comfort, cultural norms, or previous experiences, directly align with the principles of PCC [41,42]. The theory explains how respecting these preferences enhances the patient–provider relationship, improves communication, and improves patient satisfaction [43,44]. By focusing on the alignment between patient preferences and provider characteristics, PCC provides a practical foundation for analyzing how gender influences patient satisfaction and care outcomes.

The social role theory, developed by Early in 1987 and later expanded by Early and Wood in 1999 [43], explains how societal norms and cultural expectations shape individual behavior and preferences. The theory posits that traditional gender roles—culturally defined expectations about how men and women should behave—are deeply embedded in social structures [45,46]. These roles influence perceptions and preferences, including those in healthcare settings. For instance, women may be seen as nurturing and empathetic, while men may be perceived as authoritative and technically skilled. Such stereotypes often shape patient expectations and preferences for male or female healthcare providers.

The social role theory is instrumental in explaining variations in gender preferences across different cultural contexts. In conservative or patriarchal societies, where traditional gender norms are more pronounced, patients may prefer male providers for specialties requiring authority or decision making [47]. Conversely, in situations involving intimate or reproductive health, female patients may prefer female providers due to cultural notions of modesty and privacy [48]. This theory provides a sociocultural framework to understand the dynamics of gender preferences and their implications for healthcare delivery.

The integration of PCC theory and social role theory offers a robust framework for understanding the factors influencing patient preferences for provider gender. PCC focuses on aligning care delivery with patient preferences to enhance satisfaction, while social role theory contextualizes these preferences within broader societal and cultural norms. Together, these theories explain not only the existence of gender preferences but also their variation across cultural, geographical, and demographic contexts.

For example, PCC helps to analyze how respecting gender preferences can build trust and improve patient satisfaction. At the same time, social role theory provides insight into why such preferences might exist in the first place, shaped by societal values and expectations. This dual-theory approach addresses the research problem, offering a patient-centered perspective and a sociocultural understanding of gender dynamics in healthcare. By combining these theories, this study contributes to understanding how gender preferences influence patient satisfaction and offers a framework for healthcare providers and policymakers to design more inclusive, responsive, and culturally sensitive healthcare systems.

### 2.2. Methodology

**Figure 1:**
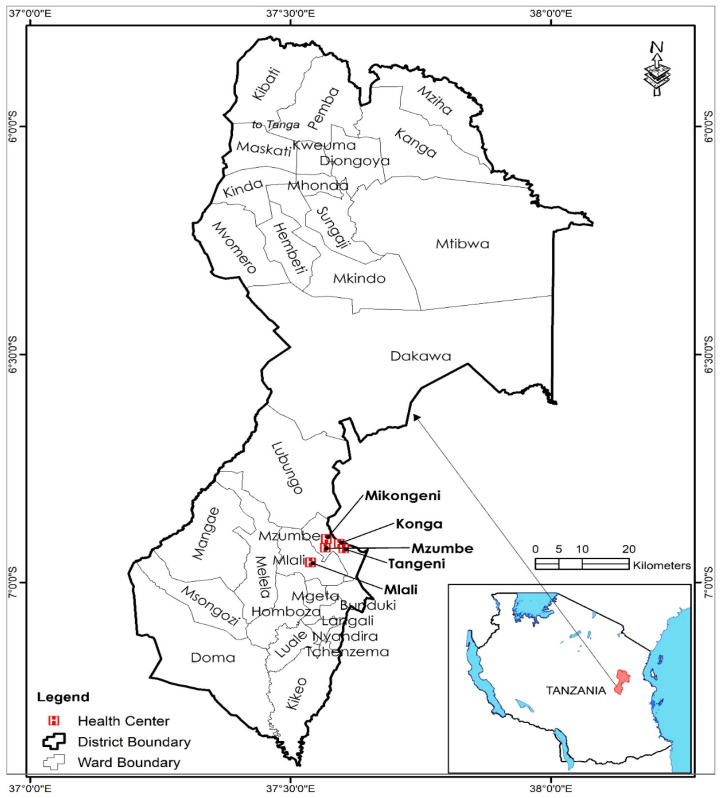
Map showing location of health centers (study areas). Source: Authors’ design using Geographic Information System (GIS) (2023).

This study was conducted in the Mvomero District of the Morogoro Region, Tanzania, focusing on five secondary-level health facilities: Mikongeni, Mzumbe, Konga, Tangeni Mission, and Mlali Health Centres (see Figure 1). These health centers offer a range of services, including general outpatient care, maternal and child health services, basic diagnostic imaging (X-ray), and specialized care in pediatrics, provided by nurses, medical doctors, X-ray personnel, and pediatricians. The facilities were selected because they represent a mix of rural and semi-urban healthcare settings, provide diverse healthcare services, and serve a wide demographic, ensuring a broader understanding of patient preferences. The Morogoro Region was chosen due to its strategic role in national health development efforts and its diverse cultural and demographic landscape, making it an ideal setting for studying variations in gender-based patient preferences. Additionally, the presence of both male and female healthcare providers across different specialties allowed for a meaningful exploration of gender dynamics in patient–provider relationships.

The study employed a cross-sectional research design, suitable for capturing data at a specific time to analyze patient preferences and their association with satisfaction levels. The positivist philosophy guided the research, emphasizing objectivity, empirical measurement, and the ability to derive generalizable findings. This approach aligns with the study’s goal of providing evidence-based insights into patient preferences. A quantitative approach is utilized to collect numerical data systematically, allowing statistical analysis to identify patterns and correlations in patient preferences and satisfaction. This methodology ensures reliable findings that inform healthcare policies and practices.

For participant selection, random sampling was employed to ensure that all eligible respondents had an equal chance of inclusion, thereby enhancing the study’s representativeness. The inclusion criteria required participants to be 18 years or older, to have accessed healthcare services at one of the selected health centers within the past six months, and to be mentally and physically capable of providing informed responses. Based on facility records, the average number of patients attending the five selected health centers over the past six months was approximately 646. The sample size was estimated using Yamane’s formula, considering this finite population and applying a 5% margin of error. Prior to the main data collection, mock interviews and a pilot study involving 20 participants were conducted to pre-test and refine the questionnaire, ensuring cultural appropriateness, clarity, and contextual relevance. The final instrument was further reviewed by academic experts to strengthen content validity. Data collection was conducted between 15 July 2023 and 16 September 2023. Out of 247 individuals approached, 240 completed the survey, yielding a high response rate of 97.16%, thus minimizing the risk of non-response bias. Ethical procedures, including obtaining ethical clearance, informed consent, and maintaining strict participant confidentiality, were rigorously observed to ensure the integrity and reliability of the research process. The sample size was calculated as follows:

$$n=\frac{N}{1+N\cdot e^{2}}=\frac{646}{1+(646\cdot 0.05^{2})}\;n=\frac{646}{2.615}=247.036\approx 247$$

### 2.3. Analytical Procedure

#### 2.3.1. Modeling Gender Preferences

A multivariate probit model was employed to analyze patient preferences regarding the gender of healthcare providers. This model is appropriate for cases where the dependent variables are binary and potentially correlated. In this study, the dependent variables indicate whether a patient expressed a preference for male providers ($Y_{1}$), female providers ($Y_{2}$), or both ($Y_{3}$), with each variable coded as 1 if the preference was selected and 0 otherwise. The model accounts for the possibility that these preferences are not independent of one another. The model can be expressed as follows:

$$UY_{i}^{*}=X\beta_{i}+\varepsilon_{i}\;for\;i=1,2,3$$

where $Y_{i}^{*}$ is the latent (unobserved) variable representing the underlying preference for provider gender i, X is a vector of explanatory variables (e.g., age, education, income, healthcare setting), $\beta_{i}$ is a vector of coefficients for the predictors for gender i, $\varepsilon_{i}$ represents the error terms, assumed to follow a multivariate normal distribution with a mean of 0 and a covariance matrix Σ.

The observed preferences ($Y_{i})$ i) are determined as follows:

$$NY_{i}=\left\{\begin{array}{c} 1\;\;if\;Y_{i}^{*}>0\;\;\\ 0\;\;Otherwise \end{array}\right.$$

The covariance matrix Σ captures the correlations between the error terms of the three equations, allowing for the interdependence of preferences. Maximum likelihood estimation was used to estimate the parameters of the model.

#### 2.3.2. Modeling Patient Satisfaction

To examine the effect of patients’ gender preferences for healthcare providers on their satisfaction, an ordered logit model (OLM) was utilized. This approach was deemed appropriate as patient satisfaction was measured on an ordinal scale with four levels: Very high satisfaction, high satisfaction, moderate satisfaction, and low satisfaction. The ordinal nature of the dependent variable indicates that the probabilities of the satisfaction levels are modeled as cumulative probabilities. Let SSS represent the satisfaction level, with the four levels coded as follows: S=1 (low satisfaction), S=2 (moderate satisfaction), S=3 (high satisfaction), and S=4 (very high satisfaction). Consequently, the probability of patient satisfaction falling into a specific category is modeled using the ordered logit framework. Therefore, the cumulative probability of patient satisfaction being less than or equal to a particular level k (k=1, 2, 3, 4) is expressed as follows:

$$P\left(S\leq k\right)=\frac{e^{\theta_{k}-X\beta }}{1+e^{\theta_{k}-X\beta }}$$

where $P\left(S\leq k\right)$ is the cumulative probability of satisfaction being at or below level k, $\theta_{k}$ are the threshold parameters to be estimated, separating the satisfaction levels, X is a vector of explanatory variables (e.g., gender of the provider, patient demographics, type of health facility), and β is a vector of coefficients associated with the explanatory variables. Thus, the probability of being in a specific satisfaction category is derived as follows:

P(S=k)=P(S≤k)−P(S≤k−1)

For k=1, P(S≤0) is defined as 0, and for k=4, P(S≤4) is defined as 1.

## 3. Results

### 3.1. Descriptive Statistics

The results in Table 1 show that slightly more than half were female (58.30%), with males accounting for 41.70%. The largest age group was 26–35 years (30.00%), followed by 36–45 years (25.00%), highlighting the predominance of young and middle-aged individuals in the sample. Most respondents had at least some formal education, with 35.00% having secondary education and 34.20% completing primary education, while 22.50% had tertiary education. Half of the respondents were married (50.00%), with 38.30% single. Farmers represented the largest occupational group (41.70%), followed by business owners (25.00%) and salaried workers (20.80%). Christians were the most represented religious group (58.30%), followed by Muslims (37.50%), reflecting the dominant faiths in the area.

Regarding healthcare utilization, Tangeni Mission Health Centre was the most visited (22.90%), while Mlali Health Centre (16.70%) was the least visited. Gender preference for healthcare providers showed a notable inclination toward male providers (42.08%), followed by female providers (30.83%), and 27.08% of respondents indicated no gender preference, expressing comfort with both male and female providers. These results emphasize the significance of gender dynamics and societal norms in shaping healthcare preferences and highlight the characteristics of individuals seeking healthcare services in the study area.

The results in Table 2 reveal notable gender differences in patients’ perceptions of care factors. Comfort with intimate care was a significant consideration, with 50.00% of respondents indicating that females were better at providing comfort in such scenarios, compared to 33.30% who preferred males. A smaller group (16.70%) found both genders equally suitable. Regarding cultural or religious norms, 41.70% believed females were more aligned with these values, while 37.50% felt males adhered better to these norms. A smaller proportion (20.80%) saw no difference, assigning equal importance to both genders.

Furthermore, the results in Table 2 show that reliability to patients was another area where gender perceptions varied. A slight majority (45.80%) indicated that females were more reliable, while 41.70% found males more dependable. Privacy and modesty, important aspects of patient care, were overwhelmingly associated with female providers, with 58.30% of respondents favoring them compared to 29.20% who preferred males. Competence in care delivery was a more divisive topic: half of the respondents (50.00%) believed males were more competent, while 33.30% assigned this attribute to females, with the remainder perceiving no difference between the genders.

When evaluating fairness and judgment, females were again rated higher (45.80%) compared to males (37.50%), though a smaller group (16.70%) believed both genders were equally critical in their judgments. For health issue-specific concerns, males were slightly favored, with 45.80% identifying them as better for handling critical health issues compared to 41.70% who preferred females. Finally, surgery ability was firmly attributed to males, with 50.00% of respondents perceiving them as better surgeons compared to 33.30% who believed females were more capable. A smaller group (16.70%) viewed both genders as equally skilled in surgical contexts. These findings highlight clear patterns of gender-based perceptions among patients, with females often viewed as more empathetic and aligned with cultural norms. At the same time, males were favored for professionalism, competence, and surgical ability. The results suggest that gender and societal expectations play critical roles in shaping patient preferences for healthcare providers.

The results in Table 3 highlight patient preferences for healthcare providers across various roles and gender-based perceptions. Most respondents (66.70%) in clinical roles believed that male and female providers were equally competent as medical doctors. However, 20.80% favored female doctors over 12.50% who preferred males. As nurses, 50.00% of respondents felt that both genders were equally proficient, while 41.70% favored female nurses compared to only 8.30% who preferred males. Similarly, 50.00% viewed both genders as equally capable pharmacists, but females (33.30%) were preferred over males (16.70%).

In administrative and support roles, a significant proportion of respondents (41.70%) believed that both genders were equally competent as health administrators, while females (33.30%) were preferred over males (25.00%). For receptionist and front desk roles, female providers were strongly favored (50.00%) over males (12.50%), with 37.50% expressing no preference. Regarding record-keeping, 45.80% preferred females compared to 16.70% who favored males, while 37.50% believed both genders were equally capable.

Moreover, the results in Table 3 further show that, in specialized roles, 41.70% of respondents felt both genders were equally skilled as emergency medical technicians (EMTs), though females (33.30%) were preferred over males (25.00%). Similarly, both genders were seen as equally competent mental health professionals by 45.80% of respondents, but females (37.50%) were more favored than males (16.70%). Interestingly, males were slightly favored in technical roles such as medical lab work, with 25.00% preferring males compared to 33.30% for females and 41.70% expressing no preference. Radiologists followed a similar pattern, with 50.00% of respondents seeing both genders as equally capable.

In support staff roles, gender-specific preferences were evident. Females were overwhelmingly preferred as housekeepers, with 62.50% of respondents favoring them compared to 12.50% for males. However, males were more strongly favored in maintenance (41.70%) and security (54.20%), highlighting perceptions of physical demands and reliability in these tasks. In IT and health informatics, 41.70% of respondents saw males as more proficient, compared to 33.30% who favored females and 25.00% who expressed no gender preference. Overall, these findings reflect gender dynamics in healthcare provider preferences. While many roles are perceived as gender-neutral, specific roles such as nursing, reception, and housekeeping are more strongly associated with females. In contrast, technical and physically demanding roles like maintenance and security are predominantly associated with males. This underlines the influence of societal norms and stereotypes in shaping patient preferences.

Table 4 presents the summary statistics of patient care factors across gender preferences, with proportions representing the mean scores and standard deviations in parentheses. Female providers received the highest ratings in several aspects of patient care, reflecting their perceived strengths in relational and empathetic care. For example, female providers were rated 4.50 (±0.70) for comfort with intimate care, 4.40 (±0.80) for perceived empathy, and 4.50 (±0.70) for privacy and modesty of patients, indicating strong patient confidence in their ability to provide personal and respectful care. While scoring lower in these areas, male providers were highly rated in technical or professional aspects such as surgery ability (4.20 ± 0.70) and perceived professionalism (4.30 ± 0.60).

Additionally, the results in Table 4 show that patients with no specific gender preference (male and female) rated providers consistently between the scores for male and female providers across most factors. For example, perceived empathy was rated 4.20 (±0.70) for both genders, lower than the rating for female providers but higher than that for male providers. Similarly, cultural or religious norms (4.00 ± 0.80) and good communication approach (4.40 ± 0.60) received balanced scores. These results suggest that while patients recognize distinct strengths associated with each gender, a significant segment values providers’ neutrality and overall competence irrespective of gender.

### 3.2. Determinants of Patients’ Preferences for Gender of Health Provider

Table 5 shows the determinants of patients’ preferences for healthcare provider gender, highlighting key factors influencing their choices. Among demographic variables, being a female patient or having a female head of household was significantly associated with a preference for male providers, with coefficients of 0.60 (*p* < 0.05) and 0.45 (*p* < 0.1), respectively. For patients preferring female providers, income emerged as a significant factor, with a coefficient of 0.35 (*p* < 0.05), suggesting that higher-income patients are more likely to select female providers. Interestingly, insurance coverage was strongly associated with all preferences, showing the highest impact on male provider preference (0.65, *p* < 0.01) and moderate but significant effects on preferences for females (0.29, *p* < 0.1) and both genders (0.40, *p* < 0.05). 

Occupational status was another significant determinant. Salaried workers were strongly associated with preferring male providers (0.70, *p* < 0.01) and moderately associated with preferences for females (0.14, *p* < 0.1) and both genders (0.45, *p* < 0.05). Business owners also leaned significantly toward male providers (0.49, *p* < 0.01) and both genders (0.19, *p* < 0.1), reflecting potential alignment with professionalism and reliability. Among religious affiliations, Christians showed significant preferences across all categories, with the highest coefficient for female providers (0.70, *p* < 0.01), suggesting that religion might influence perceptions of care dynamics.

The education level also played a significant role, with tertiary education showing strong positive associations for male providers (0.70, *p* < 0.05) and female providers (0.54, *p* < 0.01), suggesting that educated patients value diverse attributes in providers. Patients with primary education were less likely to prefer female providers (−0.55, *p* < 0.01), indicating distinct preferences based on educational background. Regarding patient health characteristics, those with infectious diseases were strongly associated with a preference for male providers (0.88, *p* < 0.01). In contrast, non-infectious diseases showed moderate effects for males (0.40, *p* < 0.05), females (0.29, *p* < 0.05), and both genders (0.35, *p* < 0.1). Younger patients (18–25 years) significantly preferred male providers (0.47, *p* < 0.01), while middle-aged patients (26–35 years) showed mild preferences for all categories.

### 3.3. Effect of Patient Care Factors on Patient Satisfaction

Table 6 presents the effects of various patient care factors on patient satisfaction, categorized into very high, high, and low satisfaction levels. Key results indicate that perceived professionalism and good communication approach had the most significant positive effects on very high satisfaction, with coefficients of 0.75 (*p* < 0.01) and 0.70 (*p* < 0.01), respectively. Similarly, perceived empathy strongly influenced high satisfaction (0.60, *p* < 0.01), emphasizing its role in enhancing patient experiences. Negative associations with low satisfaction were also evident for professionalism (−0.40, *p* < 0.1) and empathy (−0.30, *p* < 0.1), highlighting that their absence contributes to dissatisfaction.

When considering gender-specific interactions, male providers scored highly for perceived professionalism (0.86, *p* < 0.01) and privacy and modesty of patients (0.50, *p* < 0.01) in very high satisfaction. These findings suggest that male providers are valued for their professionalism and respect for patient privacy. The interaction terms for males also indicate a strong influence of perceived empathy (0.40, *p*<0.01) on high satisfaction, reinforcing the importance of relational skills for male providers in achieving higher patient satisfaction.

Female providers, on the other hand, showed strong associations with comfort with intimate care (0.50, *p* < 0.01) and perceived empathy (0.60, *p* < 0.01) in very high satisfaction. However, some factors had negative associations with female providers, such as good communication approach (−0.60, *p* < 0.01) and surgery ability (−0.55, *p* < 0.1) in very high satisfaction. These findings reflect potential biases or stereotypes that may undervalue female providers’ communication or technical skills in specific contexts.

The analysis also highlights factors influencing moderate levels of satisfaction. Cultural or religious norms were moderately associated with high satisfaction (0.40), showing that alignment with patient values plays a more supportive but less dominant role than relational or technical skills. Similarly, stereotypes about competence showed a moderate positive association with high satisfaction for both genders (0.30 for males and −0.30 for females, *p* < 0.05), suggesting that gendered perceptions of competence have a complex impact on patient satisfaction. Finally, negative associations with low satisfaction were powerful for comfort with intimate care (−0.35, *p* < 0.05) and perceived empathy (−0.30, *p* < 0.05) for female providers. These results emphasize the importance of relational care factors for female providers in avoiding patient dissatisfaction.

### 3.4. Effect of Gender Preferences on Patient Satisfaction

Table 7 shows the effects of gender preference on patient satisfaction, revealing significant patterns. Patients who preferred male providers exhibited a strong positive association with very high satisfaction (0.84, *p* < 0.01) and high satisfaction (0.47, *p* < 0.05) but a significant negative association with low satisfaction (−0.35, *p* < 0.01). This suggests that patients who prefer male providers are more likely to report higher satisfaction levels, possibly due to perceived competence or professionalism. In contrast, preferences for female providers showed a mild positive association with very high satisfaction (0.23, *p* < 0.1) and no significant effects for high or low satisfaction. These findings indicate that while male providers are more likely to elicit polarized satisfaction levels, preferences for female providers result in more neutral outcomes.

Other factors influencing satisfaction included the medical consultation fee, which was negatively associated with very high (−0.26, *p*<0.01) and high satisfaction (−0.13, *p* < 0.05) but positively associated with low satisfaction (0.24, *p*<0.01), highlighting that higher costs reduce satisfaction. Insured patients were more likely to report very high (0.32, *p* < 0.05) and high satisfaction (0.28, *p* < 0.05) but less likely to report low satisfaction (−0.30, *p* < 0.1), emphasizing the role of financial security in satisfaction levels. Patients with infectious diseases had a significant negative association with very high satisfaction (−0.60, *p* < 0.05) and high satisfaction (−0.50, *p* < 0.1), reflecting potential challenges in care delivery for these conditions. Finally, patients visiting Tangeni Mission Health Centre exhibited strong positive associations with very high (0.70, *p* < 0.01) and high satisfaction (0.55, *p* < 0.05), suggesting that facility-specific factors contribute significantly to patient satisfaction.

## 4. Discussion

This study explores how gender preferences for healthcare providers influence patients’ satisfaction, offering insights into patient–provider interactions. The findings highlight that gender preferences play a significant role, with male providers being strongly associated with very high satisfaction levels. This result aligns with existing research suggesting that male providers are often perceived as more competent in technical and procedural tasks, such as surgery and complex diagnostics [49,50]. As supported by social role theory, the positive association between male providers and patient satisfaction may reflect gendered stereotypes regarding professional authority and competence, particularly in technical fields [51]. This theory posits that societal norms shape expectations for male and female roles, explaining why male providers favor in the context of requiring authority and decisiveness [52].

In contrast, female providers were more associated with relational care factors such as empathy, comfort with intimate care, and attention to privacy and modesty. These findings are consistent with studies emphasizing the patient-centered communication styles of female providers, who perceive engaging in active listening and empathetic communication [53]. However, the relatively weaker associations between female provider preference and very high satisfaction levels suggest that relational skills, while critical, may not always outweigh technical competence in shaping patient satisfaction. This aligns with person-centered care theory, emphasizing that while relational factors are essential, patients prioritize technical competence and reliability [54,55]. Addressing this balance is essential to ensuring that both relational and technical attributes are equally valued across provider genders.

The negative associations between medical consultation fees and high satisfaction levels highlight the pervasive influence of affordability in healthcare. Patients are less satisfied when costs are perceived as burdensome, reflecting findings that financial barriers diminish trust and satisfaction [56,57,58]. Insured patients reported significantly higher satisfaction levels, reinforcing the importance of financial security in buffering against dissatisfaction. This emphasizes the need for integrating affordability and financial protections into healthcare systems to enhance satisfaction across diverse demographic groups, as supported by health equity frameworks [59,60].

Another critical finding is the variation in satisfaction levels by disease type. Patients with infectious diseases reported significantly lower satisfaction compared to those with lifestyle-related or non-infectious conditions. This disparity may stem from stigma or perceptions of complexity associated with infectious diseases, as highlighted in studies on HIV and tuberculosis care [61,62,63]. Improving care delivery for infectious diseases requires interventions that mitigate stigma and foster trust, enhancing patient satisfaction [64,65]. Efforts to incorporate tailored approaches for stigmatized conditions into facility protocols could bridge these gaps.

Facility-specific factors also emerged as crucial determinants of satisfaction, with Tangeni Mission Health Centre showing strong positive associations with very high satisfaction. This finding aligns with research emphasizing the role of organizational culture, facility reputation, and staff behavior in influencing patient experiences [66,67]. Tangeni’s positive results suggest that well-trained staff and patient-centered practices significantly enhance satisfaction. It supports Donabedian’s model of healthcare quality, which highlights the importance of structural and process attributes in delivering high-quality care [68,69].

The interaction effects between patient care factors and gender-specific preferences revealed complex dynamics, furthering our understanding of how gender perceptions shape satisfaction. Male providers were highly rated for professionalism and privacy, aligning with societal expectations that position men excelling in technical and authoritative roles [67,70]. Perception is reinforced by social role theory, which underscores the traditional association of men with assertiveness and competence in leadership domains [71,72]. For example, the strong positive interaction effects for professionalism and privacy among male providers highlight these attributes as pivotal drivers of satisfaction in contexts requiring precision and confidentiality [73,74].

On the other hand, female providers were more strongly associated with relational aspects such as empathy and comfort with intimate care, with significant interaction effects for perceived empathy. This corroborates findings from Sirera et al. [75], emphasizing the role of patient-centered communication styles in enhancing relational satisfaction. However, persistent biases against female providers in technical domains, such as surgery or advanced diagnostics, highlight systemic undervaluation of their competencies [73,76]. Addressing these biases requires healthcare systems to implement gender-sensitive training and promote equitable representation in leadership roles [69,74,77]. Educating patients about provider capabilities irrespective of gender can further mitigate stereotypes, fostering a balanced and equitable healthcare environment.

Furthermore, the influence of cultural and religious norms on gender preferences warrants deeper consideration, as these norms significantly shape patient expectations and behaviors in healthcare settings. In many traditional or religious communities, particularly within Islamic, Christian, and some conservative African societies, patients, especially women, may strongly prefer providers of the same gender due to values surrounding modesty, privacy, and moral appropriateness [78]. For example, in predominantly Muslim populations, women may avoid male healthcare providers for gynecological or reproductive health services, guided by religious teachings that emphasize gender boundaries in physical interactions. Similarly, in patriarchal societies, men may be reluctant to be treated by female providers for conditions perceived as compromising their masculinity [79]. These preferences are not simply personal but are often reinforced by family structures, community leaders, and religious teachings, influencing healthcare seeking behavior and provider trust. Ignoring these dynamics can lead to decreased satisfaction, poor communication, or delayed care, particularly in intimate or sensitive medical encounters. Therefore, understanding and integrating cultural and religious values into service delivery through gender-sensitive staffing, cultural competence training, and respectful communication are essential for improving satisfaction and ensuring equitable access to care across diverse populations.

Ultimately, these findings highlight the strong relationship between societal expectations, patient satisfaction, and healthcare delivery. They emphasize the need to integrate both relational and technical strengths across provider genders, urging healthcare systems to move beyond traditional stereotypes. Implementing gender-sensitive practices, improving affordability, and addressing disease-related stigma are critical steps toward creating an inclusive, patient-centered healthcare environment that enhances satisfaction across diverse populations. This study contributes to the existing body of knowledge by deepening the understanding of how gender preferences shape patient–provider relationships and influence satisfaction. By identifying key drivers behind these preferences, the study offers actionable insights for healthcare practitioners and policymakers. For healthcare providers, the findings can guide the development of training programs that enhance cultural competence and interpersonal skills. For policymakers, the results stress the importance of gender-sensitive recruitment and staffing strategies to align with patient preferences while promoting equity and inclusivity. Overall, these contributions aim to strengthen patient-centered care and ensure healthcare systems remain responsive to the diverse needs of their populations.

### Limitations of This Study

Despite this study’s contributions, it has several limitations that warrant consideration. Self-reported satisfaction may introduce response biases, as subjective factors and societal norms influence patient perceptions. The cross-sectional design also limits the ability to draw causal inferences between provider gender preferences and satisfaction outcomes. Furthermore, the findings are based on a specific geographic and cultural context, which may restrict their generalizability to other settings with differing healthcare systems and cultural dynamics. Furthermore, research should employ longitudinal designs to track changes in satisfaction over time and explore the mechanisms underlying gender preferences to address these limitations. Expanding studies to diverse cultural and geographic contexts would enhance the applicability of findings, while qualitative methods could provide deeper insights into patient experiences. Finally, interventions targeting gender biases and improving provider competencies should be evaluated to determine their impact on satisfaction outcomes.

## 5. Conclusions

This study highlights the critical role of gender preferences and patient care factors in shaping patient satisfaction. Male providers were strongly associated with technical competence and professionalism, while female providers excelled in relational care aspects such as empathy and comfort. These findings emphasize the interplay between societal gender norms and healthcare delivery, revealing how perceptions of provider gender can influence patient satisfaction. Significantly, satisfaction was also affected by factors such as affordability, insurance coverage, and facility-specific attributes, suggesting that structural and systemic issues significantly impact patient experiences.

These findings can be grounded firmly within social role theory and patient-centered care (PCC) theory. Social role theory provides a clear explanation of how societal expectations and cultural norms influence patient preferences, resulting in distinct gender-based perceptions of provider competence, with men often perceived as technically skilled and women seen as empathetic caregivers. Similarly, the patient-centered care theory underscores the importance of respecting patient preferences to enhance trust, effective communication, and patient satisfaction. Integrating these theoretical frameworks helps clarify not only the presence of gender-based preferences in healthcare but also their sociocultural origins, providing a foundation for developing more inclusive and responsive healthcare practices.

The results have important implications for health policy. Policymakers should prioritize initiatives aimed at challenging gender stereotypes within healthcare systems. Public awareness campaigns can educate patients on provider competencies, emphasizing that skills in technical and relational care are not inherently gender specific. Health policies should also mandate comprehensive training in communication and patient relations for healthcare professionals, ensuring all providers demonstrate empathy, compassion, and effective interpersonal skills regardless of gender. Additionally, policies addressing affordability and equitable access, such as expanded insurance coverage, transparent pricing structures, and targeted subsidies, should be implemented to reduce financial barriers and improve overall patient satisfaction.

From a clinical practice perspective, healthcare managers should strategically use these insights to enhance service delivery. Facilities should implement continuous professional development programs emphasizing both technical and relational skills training for all healthcare providers. Recruitment and staff assignment strategies might also consider patient preferences regarding provider gender, particularly in specialties dealing with sensitive issues such as reproductive health, psychological counseling, and chronic illnesses. Furthermore, facility managers should systematically evaluate patient satisfaction, incorporating feedback mechanisms that explicitly explore patient–provider gender interactions. Furthermore, health administrators can proactively tailor clinical environments to be more patient centered, culturally sensitive, and responsive to diverse patient expectations.

## Figures and Tables

**Table 1:** General characteristics of respondents (patient).

Demographic Variable	Categories	Frequency (*n*)	Percentage (%)
Sex	Male	100	41.70%
	Female	140	58.30%
Age group	18–25 years	40	16.70%
	26–35 years	72	30.00%
	36–45 years	60	25.00%
	46–55 years	45	18.80%
	Above 55 years	23	9.60%
Education level	No formal education	20	8.30%
	Primary education	82	34.20%
	Secondary education	84	35.00%
	Tertiary education	54	22.50%
Marital status	Single	92	38.30%
	Married	120	50.00%
	Divorced/separated	18	7.50%
	Widowed	10	4.20%
Occupation	Farmer	100	41.70%
	Business owner	60	25.00%
	Salaried worker	50	20.80%
	Unemployed	30	12.50%
Religion	Christian	140	58.30%
	Muslim	90	37.50%
	Other	10	4.20%
Healthcare facility visited	Mikongeni Health Centre	50	20.80%
	Mzumbe Health Centre	50	20.80%
	Konga Health Centre	45	18.80%
	Tangeni Mission Health Centre	55	22.90%
	Mlali Health Centre	40	16.70%
Health provider gender preference	Male	101	42.08%
	Female	74	30.83%
	Both male and female	65	27.08%

**Table 2:** Patient preferences for healthcare provider attributes by gender (*n* = 240).

	Differences Across Gender Preferences	Overall Response	Responses Based on Gender	
			Male	Female
Comfort with Intimate Care	Males treat by comfort with intimate care	80 (33.30%)	50 (20.80%)	30 (12.50%)
	Females treat by comfort with intimate care	120 (50.00%)	30 (12.50%)	90 (37.50%)
	Both males and females treat by comfort	40 (16.70%)	20 (8.30%)	20 (8.30%)
Cultural or Religious Norms	Males are more cultural and religious	90 (37.50%)	50 (20.80%)	40 (16.70%)
	Females are more cultural and religious	100 (41.70%)	30 (12.50%)	70 (29.20%)
	Both males and females are cultural and religious	50 (20.80%)	20 (8.30%)	30 (12.50%)
Perceived Empathy	Males are more empathetic	60 (25.00%)	40 (16.70%)	20 (8.30%)
	Females are more empathetic	140 (58.30%)	30 (12.50%)	110 (45.80%)
	Both males and females are empathetic	40 (16.70%)	20 (8.30%)	20 (8.30%)
Perceived Professionalism	Males are more professional	110 (45.80%)	64 (26.70%)	46 (19.20%)
	Females are more professional	90 (37.50%)	30 (12.50%)	60 (25.00%)
	Both males and females are professional	40 (16.70%)	20 (8.30%)	20 (8.30%)
Good Communication Approach	Males have good communication approach	80 (33.30%)	50 (20.80%)	30 (12.50%)
	Females have good communication approach	120 (50.00%)	30 (12.50%)	90 (37.50%)
	Both males and females have good communication	40 (16.70%)	20 (8.30%)	20 (8.30%)
Reliability to Patients	Males are more reliable to patients	100 (41.70%)	60 (25.00%)	40 (16.70%)
	Females are more reliable to patients	110 (45.80%)	30 (12.50%)	80 (33.30%)
	Both males and females are reliable	30 (12.50%)	20 (8.30%)	10 (4.20%)
Privacy and Modesty of Patients	Males prioritize privacy and modesty	70 (29.20%)	50 (20.80%)	20 (8.30%)
	Females prioritize privacy and modesty	140 (58.30%)	30 (12.50%)	110 (45.80%)
	Both males and females prioritize privacy	30 (12.50%)	20 (8.30%)	10 (4.20%)
Stereotypes about Competence	Males are more competent	120 (50.00%)	64 (26.70%)	56 (23.30%)
	Females are more competent	80 (33.30%)	30 (12.50%)	50 (20.80%)
	Both males and females are competent	40 (16.70%)	20 (8.30%)	20 (8.30%)
Fairness and Judgement	Males are fair and critical in judgement	90 (37.50%)	60 (25.00%)	30 (12.50%)
	Females are fair and critical in judgement	110 (45.80%)	30 (12.50%)	80 (33.30%)
	Both males and females are critical	40 (16.70%)	20 (8.30%)	20 (8.30%)
Health Issue-Specific Concerns	Males are good for critical health issues	110 (45.80%)	60 (25.00%)	50 (20.80%)
	Females are good for critical health issues	100 (41.70%)	30 (12.50%)	70 (29.20%)
	Both males and females are good for issues	30 (12.50%)	20 (8.30%)	10 (4.20%)
Surgery Ability	Males are good in surgery	120 (50.00%)	64 (26.70%)	56 (23.30%)
	Females are good in surgery	80 (33.30%)	30 (12.50%)	50 (20.80%)
	Both males and females are good in surgery	40 (16.70%)	20 (8.30%)	20 (8.30%)

**Table 3:** Patient preferences for healthcare providers by gender and professional category (*n* = 240).

Health Providers	Differences Across Gender	Overall Response	Male	Female
Clinical staff	Males are better medical doctors	30 (12.50%)	20 (8.30%)	10 (4.20%)
	Females are better medical doctors	50 (20.80%)	15 (6.30%)	35 (14.60%)
	Both males and females are better medical doctors	160 (66.70%)	80 (33.30%)	80 (33.30%)
	Males are better nurses	20 (8.30%)	15 (6.30%)	5 (2.10%)
	Females are better nurses	100 (41.70%)	40 (16.70%)	60 (25.00%)
	Both males and females are better nurses	120 (50.00%)	65 (27.10%)	55 (22.90%)
	Males are better pharmacists	40 (16.70%)	25 (10.40%)	15 (6.30%)
	Females are better pharmacists	80 (33.30%)	30 (12.50%)	50 (20.80%)
	Both males and females are better pharmacists	120 (50.00%)	60 (25.00%)	60 (25.00%)
Administrative staff	Males are better health administrators	60 (25.00%)	40 (16.70%)	20 (8.30%)
	Females are better health administrators	80 (33.30%)	30 (12.50%)	50 (20.80%)
	Both males and females are better health administrators	100 (41.70%)	50 (20.80%)	50 (20.80%)
	Males are better receptionists and front desk officers	30 (12.50%)	20 (8.30%)	10 (4.20%)
	Females are better receptionists and front desk officers	120 (50.00%)	50 (20.80%)	70 (29.20%)
	Both males and females are better receptionists and front desk officers	90 (37.50%)	45 (18.80%)	45 (18.80%)
	Males are better at record keeping	40 (16.70%)	30 (12.50%)	10 (4.20%)
	Females are better at record keeping	110 (45.80%)	45 (18.80%)	65 (27.10%)
	Both males and females are better at record keeping	90 (37.50%)	45 (18.80%)	45 (18.80%)
Specialized roles	Males are better therapists	50 (20.80%)	35 (14.60%)	15 (6.30%)
	Females are better therapists	100 (41.70%)	40 (16.70%)	60 (25.00%)
	Both males and females are better therapists	90 (37.50%)	45 (18.80%)	45 (18.80%)
	Males are better emergency medical technicians (EMTs)	60 (25.00%)	40 (16.70%)	20 (8.30%)
	Females are better emergency medical technicians (EMTs)	80 (33.30%)	35 (14.60%)	45 (18.80%)
	Both males and females are better emergency medical technicians (EMTs)	100 (41.70%)	50 (20.80%)	50 (20.80%)
	Males are better mental health professionals	40 (16.70%)	25 (10.40%)	15 (6.30%)
	Females are better mental health professionals	90 (37.50%)	35 (14.60%)	55 (22.90%)
	Both males and females are better mental health professionals	110 (45.80%)	55 (22.90%)	55 (22.90%)
Technical staff	Males are better in medical labs	60 (25.00%)	40 (16.70%)	20 (8.30%)
	Females are better in medical labs	80 (33.30%)	35 (14.60%)	45 (18.80%)
	Both males and females are better in medical labs	100 (41.70%)	50 (20.80%)	50 (20.80%)
	Males are better radiologists	50 (20.80%)	35 (14.60%)	15 (6.30%)
	Females are better radiologists	70 (29.20%)	30 (12.50%)	40 (16.70%)
	Both males and females are better radiologists	120 (50.00%)	60 (25.00%)	60 (25.00%)
Support staff	Males are better housekeepers	30 (12.50%)	20 (8.30%)	10 (4.20%)
	Females are better housekeepers	150 (62.50%)	50 (20.80%)	100 (41.70%)
	Both males and females are better housekeepers	60 (25.00%)	30 (12.50%)	30 (12.50%)
	Males are better in maintenance	100 (41.70%)	60 (25.00%)	40 (16.70%)
	Females are better in maintenance	50 (20.80%)	20 (8.30%)	30 (12.50%)
	Both males and females are better in maintenance	90 (37.50%)	45 (18.80%)	45 (18.80%)
	Males are better in security	130 (54.20%)	80 (33.30%)	50 (20.80%)
	Females are better in security	40 (16.70%)	15 (6.30%)	25 (10.40%)
	Both males and females are better in security	70 (29.20%)	35 (14.60%)	35 (14.60%)
Information technology (IT) and health informatics	Males are better in IT and health informatics	100 (41.70%)	70 (29.20%)	30 (12.50%)
	Females are better in IT and health informatics	80 (33.30%)	30 (12.50%)	50 (20.80%)
	Both males and females are better in IT and health informatics	60 (25.00%)	30 (12.50%)	30 (12.50%)

**Table 4:** Summary statistics by gender preferences.

Patient Care Factors	Male	Female	Both Male and Female
Comfort with Intimate Care	4.11 (0.80)	4.50 (0.70)	4.30 (0.6)
Cultural or Religious Norms	3.80 (1.00)	4.20 (0.90)	4.00 (0.80)
Perceived Empathy	4.00 (0.90)	4.40 (0.80)	4.20 (0.70)
Perceived Professionalism	4.30 (0.60)	4.60 (0.50)	4.50 (0.50)
Good Communication Approach	4.20 (0.70)	4.50 (0.60)	4.40 (0.60)
Reliability to Patients	4.11 (0.80)	4.30 (0.70)	4.20 (0.70)
Privacy and Modesty of Patients	4.00 (0.90)	4.50 (0.70)	4.30 (0.70)
Stereotypes about Competence	3.90 (1.00)	4.30 (0.80)	4.21 (0.90)
Fairness and Judgement	4.10 (0.80)	4.40 (0.70)	4.30 (0.70)
Health Issue-Specific Concerns	3.80 (1.00)	4.20 (0.90)	4.00 (0.80)
Surgery Ability	4.20 (0.70)	4.40 (0.60)	4.30 (0.60)

Reference: Standard deviations in parentheses.

**Table 5:** Determinant of patients’ preferences for health provider gender.

	Gender Preferences		
	Males	Females	Both (Males and Females)
Female patient head of household	0.45 * (0.12)	0.04 (0.15)	0.18 (0.11)
Sex of patient (female)	0.60 ** (0.14)	0.10 (0.18)	0.18 (0.13)
Patient household size	−0.10 (0.23)	−0.08 (0.08)	0.05 (0.06)
Patient income	0.20 * (0.07)	0.35 ** (0.08)	0.15 (0.05)
Medical consultation fee	−0.15 ** (0.02)	−0.27 (0.23)	−0.03 (0.02)
Insured patient	0.65 *** (0.13)	0.29 * (0.10)	0.40 ** (0.12)
**Patient Occupation (ref: unemployed)**			
Farmer	0.38 ** (0.10)	0.16 * (0.07)	0.13 * (0.05)
Business owner	0.49 *** (0.01)	0.05 (0.13)	0.19 * (0.09)
Salaried worker	0.70 *** (0.12)	0.14 * (0.04)	0.45 ** (0.09)
**Patient Religion (ref: other religion)**			
Christian	0.50 ** (0.12)	0.70 *** (0.10)	0.40 ** (0.09)
Muslim	0.35 (0.09)	0.13 (0.08)	0.25 (0.18)
**Patient-visited health centers (ref: Mlali Health Centre)**			
Mikongeni Health Centre	0.40 ** (0.10)	0.01 (0.12)	0.01 (0.09)
Mzumbe Health Centre	0.45 ** (0.11)	0.15 ** (0.03)	0.09 ** (0.01)
Konga Health Centre	0.50 (0.12)	0.70 (0.14)	0.40 (0.11)
Tangeni Mission Health Centre	0.37 *** (0.03)	0.80 *** (0.15)	0.45 ** (0.12)
**Patient education (ref: no formal education)**			
Primary education	0.35 ** (0.08)	−0.55 *** (0.10)	0.30 (0.27)
Secondary education	0.45 ** (0.09)	0.15 * (0.06)	0.05 (0.08)
Tertiary education	0.70 ** (0.10)	0.54 *** (0.12)	0.20 ** (0.09)
**Patient marital status (ref: widowed)**			
Single	0.40 *** (0.00)	0.03 (0.12)	0.30 (0.09)
Married	0.27 * (0.11)	0.18 (0.13)	0.09 (0.10)
Divorced/separated	0.30 (0.20)	0.50 (0.31)	0.25 (0.28)
**Patient age (ref:** above 55 years)			
18–25 years	0.47 *** (0.08)	0.19 (0.10)	0.15 (0.27)
26–35 years	0.35 *** (0.09)	0.15 * (0.06)	0.09 * (0.01)
36–45 years	0.50 (0.10)	0.70 (0.12)	0.35 (0.09)
46–55 years	0.40 (0.10)	0.60 (0.12)	0.30 (0.09)
**Patient diseases (ref: non-infectious diseases—hereditary)**			
Infectious diseases	0.88 *** (0.03)	0.29 * (0.11)	0.20 (0.12)
Non-infectious diseases	0.40 ** (0.19)	0.29 ** (0.08)	0.35 * (0.11)

* *p* < 0.1 (indicates results that are significant at 10% level), ** *p* < 0.05 (indicates results that are significant at 5% level), *** *p* < 0.01 (indicates results that are significant at 1% level); standard errors in parentheses.

**Table 6:** Effects of patient care factors on patient satisfaction (ref: moderate satisfaction).

.	Very High Satisfaction	High Satisfaction	Low Satisfaction
Comfort with Intimate Care	0.45 * (0.20)	0.35 (0.28)	−0.20 (0.17)
Cultural or Religious Norms	0.50 (0.32)	0.40 (0.29)	0.25 (0.16)
Perceived Empathy	0.60 *** (0.10)	0.48 ** (0.21)	−0.30 * (0.14)
Perceived Professionalism	0.75 *** (0.14)	0.45 ** (0.11)	−0.40 * (0.17)
Good Communication Approach	0.70 *** (0.12)	0.25 * (0.09)	−0.25 (0.14)
Reliability to Patients	0.53 *** (0.19)	0.19 * (0.09)	−0.30 (0.23)
Privacy and Modesty of Patients	0.25 *** (0.03)	0.10 ** (0.03)	−0.35 (0.22)
Stereotypes about Competence	0.40 (0.28)	0.30 (0.18)	−0.20 (0.23)
Fairness and Judgement	0.22 (0.15)	0.23 (0.19)	−0.25 (0.18)
Health Issue-Specific Concerns	0.50 (0.42)	0.35 (0.19)	−0.30 (0.21)
Surgery Ability	0.60 *** (0.10)	0.24 * (0.10)	−0.06 * (0.01)
Comfort with Intimate Care*Male	0.30 ** (0.09)	0.15 * (0.07)	0.01 (0.08)
Cultural or Religious Norms*Male	0.35 (0.10)	0.30 (0.38)	0.20 (0.39)
Perceived Empathy*Male	0.40 *** (0.01)	0.34 (0.29)	0.05 (0.26)
Perceived Professionalism*Male	0.86 *** (0.12)	0.45 ** (0.10)	−0.30 ** (0.09)
Good Communication Approach*Male	0.45 (0.30)	0.40 (0.26)	0.05 (0.08)
Reliability to Patients*Male	0.40 (0.29)	0.35 (0.39)	0.20 (0.19)
Privacy and Modesty of Patients*Male	0.50 *** (0.12)	0.40 ** (0.10)	0.14 (0.11)
Stereotypes about Competence*Male	0.30 *** (0.00)	0.25 ** (0.08)	0.15 (0.08)
Fairness and Judgement*Male	0.40 (0.22)	0.35 (0.19)	0.05 (0.10)
Health Issue-Specific Concerns*Male	0.35 ** (0.11)	0.30 (0.16)	0.07 (0.10)
Surgery Ability*Male	0.40 ** (0.12)	0.35 (0.17)	0.22 (0.18)
Comfort with Intimate Care*Female	0.50 *** (0.13)	0.40 ** (0.10)	−0.35 ** (0.12)
Cultural or Religious Norms*Female	0.55 (0.44)	0.45 (0.31)	−0.40 (0.23)
Perceived Empathy*Female	0.60 *** (0.15)	0.50 ** (0.12)	0.02 (0.17)
Perceived Professionalism*Female	−0.17 ** (0.04)	−0.08 * (0.02)	0.13 (0.10)
Good Communication Approach*Female	−0.60 *** (0.14)	−0.50 ** (0.11)	0.45 (0.23)
Reliability to Patients*Female	−0.57 ** (0.13)	−0.08 (0.10)	0.40 (0.32)
Privacy and Modesty of Patients*Female	0.25 (0.15)	0.15 (0.12)	0.07 (0.14)
Stereotypes about Competence*Female	−0.40 ** (0.11)	−0.30 ** (0.09)	0.04 (0.10)
Fairness and Judgement*Female	−0.32 (0.12)	−0.26 (0.10)	0.19 (0.11)
Health Issue-Specific Concerns*Female	−0.45 * (0.20)	−0.35 (0.27)	0.15 (0.19)
Surgery Ability*Female	−0.55 * (0.23)	−0.45 * (0.21)	0.36 (0.22)
Patient demographic controls	Yes	Yes	Yes

* *p* < 0.1, ** *p* < 0.05, *** *p* < 0.01; standard errors in parentheses.

**Table 7:** Effects of gender preference on patient satisfaction.

	Very High Satisfaction	High Satisfaction	Low Satisfaction
**Gender preference (ref: both male and female)**			
Male	0.84 *** (0.02)	0.47 ** (0.10)	−0.35 *** (0.01)
Female	0.23 * (0.09)	0.09 (0.06)	0.09 (0.10)
Medical consultation fee	−0.26 *** (0.02)	−0.13 ** (0.02)	0.24 *** (0.02)
Insured patient	0.32 ** (0.13)	0.28 ** (0.08)	−0.30 * (0.12)
**Patient diseases (ref: non-infectious diseases—hereditary)**			
Infectious diseases	−0.60 ** (0.24)	−0.50 * (0.22)	0.08 (0.13)
Non-infectious diseases (lifestyle)	0.20 * (0.09)	0.16 (0.11)	−0.07 (0.11)
**Patient visited health centres (ref: Mlali Health Centre)**			
Mikongeni Health Centre	−0.24 (0.13)	−0.18 (0.10)	0.35 (0.21)
Mzumbe Health Centre	0.21 (0.13)	0.45 * (0.21)	−0.40 (0.12)
Konga Health Centre	−0.06 (0.14)	−0.13 (0.10)	0.41 (0.22)
Tangeni Mission Health Centre	0.70 *** (0.15)	0.55 ** (0.13)	−0.04 (0.14)
Patient demographic controls	Yes	Yes	Yes
Patient care factors control	No	No	No
Observations	240		
LR Chi squared (12)	37.08		
Prob > Chi squared	48.13		
Pseudo R squared	0.3564		

* *p* < 0.1, ** *p* < 0.05, *** *p* <0.01; standard errors in parentheses.

## Data Availability

All the data are provided within the paper. Moreover, data files can be shared on request through email.
